# Hypocoercivity and Fast Reaction Limit for Linear Reaction Networks with Kinetic Transport

**DOI:** 10.1007/s10955-020-02503-5

**Published:** 2020-02-14

**Authors:** Gianluca Favre, Christian Schmeiser

**Affiliations:** Faculty of Mathematics, University of Vienna, Oskar-Morgenstern-Platz 1, 1090 Wien, Austria

**Keywords:** Hypocoercivity, Kinetic-reaction equation, Kinetic transpor, Fast reaction limit

## Abstract

The long time behavior of a model for a first order, weakly reversible chemical reaction network is considered, where the movement of the reacting species is described by kinetic transport. The reactions are triggered by collisions with a nonmoving background with constant temperature, determining the post-reactional equilibrium velocity distributions. Species with different particle masses are considered, with a strong separation between two groups of light and heavy particles. As an approximation, the heavy species are modeled as nonmoving. Under the assumption of at least one moving species, long time convergence is proven by hypocoercivity methods for the cases of positions in a flat torus and in whole space. In the former case the result is exponential convergence to a spatially constant equilibrium, and in the latter it is algebraic decay to zero, at the same rate as solutions of parabolic equations. This is no surprise since it is also shown that the macroscopic (or reaction dominated) behavior is governed by the diffusion equation.

## Introduction

We consider *N* chemical species $S_1,\ldots , S_N$ with different particle masses moving in a periodic box or in whole space. The interaction with a stationary background with constant temperature *T* triggers first order chemical reactions with reaction rates independent of the velocity of the incoming particle. The velocity of the outgoing particle is sampled from a Maxwellian distribution with parameters taken from the background, i.e. mean velocity zero and temperature *T*. The resulting reaction network is assumed to be connected and weakly reversible, meaning that for each reaction $S_i\rightarrow S_j$ there exists a reaction path $S_j \rightarrow \cdots \rightarrow S_i$.

These assumptions lead to a system of *N* linear kinetic transport equations for the phase space number densities of the reacting species. We shall make the additional assumption that the species can be split into two groups of *light* and *heavy* particles, where the particle masses are of comparable size within each group but strongly disparate between the groups. A corresponding nondimensionalization of the equations, assuming at least one light species, will suggest a simplified model, where the heavy particles do not move. As a result we consider a system of kinetic equations (for the light species) coupled, via the reaction terms, to a system of ordinary differential equations (pointwise in position space, for the heavy species).

The construction of equilibrium solutions is straightforward. In equilibrium, the position densities are constant, and the velocity distributions of the light particles are Maxwellians. The position densities are complex balanced equilibria of the reaction network. Existence and uniqueness for given total mass are standard results of the theory of chemical reaction networks.

Our main results are exponential convergence to equilibrium in the case of the periodic box and algebraic decay to zero in whole space. In both situations the rates and constants are computable. Although general results for Markov processes imply that relative entropies are nonincreasing [10], the decay result is not obvious, since the entropy dissipation is not coercive relative to the equilibrium. We employ the abstract $L^2$-hypocoercivity method of [6, 7] and its extension to whole space problems [2]. The main difficulty is the proof of *microscopic coercivity,* meaning here that the reaction terms without the transport produce exponential convergence to a *local equilibrium,* where the total number density of all species might still depend on position and time. Two alternative proofs are presented. In the first one, relaxation in velocity space is separated from relaxation to chemical equilibrium and known results for the latter [8] could be used. The second proof extends the proof in [8] by introducing reaction paths in species-velocity space. For completeness and comparability we fully present both proofs, showing that the second proof never gives a worse result.

The second result is a macroscopic or fast-reaction limit. For length scales large compared to the mean free path between reaction events and for the corresponding diffusion time scales, the system is in local equilibrium and the total number density solves the heat equation.

Systematic approaches to hypocoercivity have been started in [15, 18], where Lyapunov functions based on modified $H^1$-norms are constructed. More recently, an approach without smoothness assumptions on initial data, motivated by [11], has been developed in [6, 7], see [5, 14] for overviews. Recently the latter approach has been extended to the analysis of algebraic decay rates in whole space problems [2]. Hypocoercivity for systems of kinetic equations coupled by linearized collision terms has been shown in [4]. For a nonlinear system modeling a second order pair generation-recombination reaction, both hypocoercivity and the fast reaction limit have been analyzed in [17].

This work can be seen as an extension of the corresponding result for linear reaction diffusion models [8], which has recently been extended to general mass action kinetics [9], bringing the theory for reaction diffusion models close to the best results on the global attractor conjecture [13] for ODE models without transport [3].

Many extensions of the present results are desirable. Besides the inclusion of collision effects and of second order reactions, questions of energy and momentum balance pose significant challenges, where a trade-off between mathematical manageability and modeling precision has to be found. One goal is the rigorous justification of the derivation of reaction diffusion systems from kinetic models as an extension of results for linear cases [1].

Finally, we describe the structure of the rest of this article. In the following section the kinetic model is formulated including a dimensional analysis and the reduction to a system with partially nonmoving species. The formal macroscopic limit is presented and our main results on the long term behavior of solutions and on the rigorous justification of the macroscopic limit are formulated. In Sect. 3 our main technical result on ’microscopic coercivity’ is proven, i.e. a spectral gap for the reaction operator. Sections 4 and 5 are concerned with the proofs of our main results on long time behaviour and, respectively, on the rigorous macroscopic limit.

## The Model—Main Results

We denote the chemical species by $S_1,\dots ,S_N$ and the reaction constant for the reaction $S_i\rightarrow S_j$ by $k_{ji}\ge 0$, $i,j=1,\ldots ,N$, where $k_{ji}=0$ means that the reaction does not occur. More completely, also including velocities $v\in {\mathbb {R}}^d$, we assume that the jump $(S_i,v) \rightarrow (S_j,v')$ occurs with rate constant $k_{ji}M_j(v')$, as described above independent of the incoming velocity, where the Maxwellian distribution is given by

$$\begin{aligned} M_i(v) = \bigg (\frac{2 \pi k_B T}{m_i}\bigg )^{-d/2} \exp \bigg (- \frac{|v|^2 m_i}{2 k_B T} \bigg ) \,, \end{aligned}$$

with the Boltzmann constant $k_B$, the constant given background temperature *T*, and the particle masses $m_1\le \cdots \le m_N$ of the respective species $S_1,\ldots ,S_N$. Actually all our results can be proven with $M_1,\ldots ,M_N$ replaced by arbitrary probability distributions with mean zero and finite fourth order moments.

The phase space number density of species $S_i$ at time $t\ge 0$ is denoted by $f_i(x,v,t)\ge 0$, $i=1,\ldots ,N$, with the position variable *x*. We consider two cases:

1. *Periodic box:*$x\in {\mathbb {T}}^d$, the flat *d*-dimensional torus, represented by the cube $[0,L]^d$ with periodic boundary conditions for $f_1,\ldots ,f_N$.
2. *Whole space:*$x\in {\mathbb {R}}^d$, with $f_1,\ldots ,f_N$ integrable, i.e. a finite total number of particles.

In the following, integrations with respect to *x* will be written over $\Omega$, where $\Omega = [0,L]^d$ for the periodic box and $\Omega = {\mathbb {R}}^d$ for whole space.

The phase space distributions satisfy the evolution system

1 $$\begin{aligned} \partial _t f_i + v \cdot \nabla _xf_i = \sum _{j=1}^{N}\big (k_{ij}\rho _j M_i - k_{ji}f_i\big ) \,,\qquad i=1,\ldots ,N\,, \end{aligned}$$

where the left hand side describes free transport and the right hand side the chemical reactions with position densities

2 $$\begin{aligned} \rho _j(x,t) = \int _{{\mathbb {R}}^d}f_j(x,v,t)\,dv\; \,, \end{aligned}$$

where we will sometimes also use the notation $\rho _{f,j}$ to avoid ambiguity. We assume that there are $N_l\ge 1$ light species $S_1,\ldots ,S_{N_l}$ and $N-N_l$ heavy species $S_{N_l+1},\ldots ,S_N$. The separation of the two groups is expressed in the assumption

$$\begin{aligned} \mu := \frac{m_{N_l}}{m_{N_l+1}} \ll 1 \,. \end{aligned}$$

In a nondimensionalization we introduce as reference velocity the thermal velocity

$$\begin{aligned} v_{th} := \sqrt{\frac{k_B T}{m_{N_l}}} \end{aligned}$$

of the heaviest light species $S_{N_l}$. As reference time ${\overline{t}}$ we choose an average value of $k_{ij}^{-1}$, $i,j = 1,\ldots , N$. The reference length is given by ${\overline{x}} = {\overline{t}} \,v_{th}$. After the nondimensionalization

$$\begin{aligned}&v \rightarrow v_{th} v \,,\quad t \rightarrow {\overline{t}} t \,,\quad x \rightarrow \overline{x} x \,,\quad f_i \rightarrow ({\overline{x}} v_{th})^{-d} f_i \,,\quad M_i \rightarrow v_{th}^{-d} M_i\,, \quad \\&\qquad k_{ij} \rightarrow \frac{k_{ij}}{{\overline{t}}} \,,\quad L \rightarrow {\overline{x}} L \,, \end{aligned}$$

the equations (1), (2) look the same, but with

3 $$\begin{aligned} M_i(v) = \left( 2 \pi \theta _i\right) ^{-d/2} \exp \left( - \frac{|v|^2}{2\theta _i} \right) \,,\qquad \theta _i = \frac{m_{N_l}}{m_i} \,, \qquad i = 1,\ldots ,N\,. \end{aligned}$$

In particular we have $\theta _i \ge 1$, $i=1,\ldots ,N_l$, for the light particles and $\theta _i = O(\mu )$, $i=N_l + 1,\ldots ,N$, for the heavy particles, such that $M_i(v) \rightarrow \delta (v)$, $i=N_l + 1,\ldots ,N$, in the distributional sense as $\mu \rightarrow 0$. In this limit it is consistent to also look for solutions, where the heavy particles are nonmoving, i.e. $f_i(x,v,t) = \rho _i(x,t)\delta (v)$, $i=N_l + 1,\ldots ,N$. Therefore, for the rest of this work we shall consider the system

4 $$\begin{aligned} \partial _t f_i + v \cdot \nabla _xf_i= & {} \sum _{j=1}^{N}\big (k_{ij}\rho _j M_i - k_{ji}f_i\big ) \,,\qquad i=1,\ldots ,N_l\,, \end{aligned}$$

5 $$\begin{aligned} \partial _t \rho _i= & {} \sum _{j=1}^{N}\big (k_{ij}\rho _j - k_{ji}\rho _i\big ) \,,\qquad i=N_l+1,\ldots ,N\,, \end{aligned}$$

with $N_l\ge 1$ and with $M_i$ given by (3), subject to initial conditions

6 $$\begin{aligned} f_i(x,v,0) = f_{I,i}(x,v) \,,\quad \rho _j(x,0) = \rho _{I,j}(x) \,,\quad x\in \Omega \,,\, v\in {\mathbb {R}}^d\,,\, i \le N_l\,,\, j > N_l \,, \end{aligned}$$

with initial data satisfying $f_{I,i}\in L_+^1(\Omega \times {\mathbb {R}}^d)$, $\rho _{I,j}\in L_+^1(\Omega )$. For simplicity of notation we formally set $f_i(x,v,t) = \rho _i(x,t)M_i(v)$ with $M_i(v) = \delta (v)$, $i> N_l$, and write the system (4), (5) in the equivalent form

7 $$\begin{aligned} \partial _t f_i + \sigma _i \,v \cdot \nabla _xf_i = \sum _{j=1}^{N}\big (k_{ij}\rho _j M_i - k_{ji}f_i\big ) \,,\qquad i=1,\ldots ,N\,, \end{aligned}$$

with $\sigma _i = 1$ for $i\le N_l$ and $\sigma _i = 0$ otherwise. We shall consider initial value problems with

8 $$\begin{aligned} f(x,v,0) = f_I(x,v) \,,\qquad x\in \Omega \,,\quad v\in {\mathbb {R}}^d \,. \end{aligned}$$

The system (7) conserves the total number of particles: The total position density

$$\begin{aligned} \rho (x,t) := \sum _{i=1}^N \rho _i(x,t) \end{aligned}$$

satisfies

9 $$\begin{aligned} \partial _t \rho + \nabla _x\cdot \left( \sum _{i=1}^{N_l} \int _{{\mathbb {R}}^d} v f_i \,dv\right) = 0 \,, \end{aligned}$$

and therefore

$$\begin{aligned} \int _{\Omega } \rho (x,t)dx = {\mathbf{M }} := \sum _{i=1}^{N_l} \int _{\Omega } \int _{{\mathbb {R}}^d} f_{I,i}(x,v) dv\,dx + \sum _{j=N_l+1}^N \int _\Omega \rho _{I,j}(x)dx \,,\qquad t\ge 0\,. \end{aligned}$$

### Local and Global Equilibria

#### Definition 1

(Local equilibrium) A state $f(x,v,t)=(f_1(x,v,t), \ldots , f_N(x,v,t))$ is called a local equilibrium for (7), if it balances the reactions, i.e., if

10 $$\begin{aligned} \sum _{j=1}^{N}\big (k_{ij}\rho _j M_i - k_{ji}f_i\big ) =0\,,\qquad i=1,\ldots ,N\,. \end{aligned}$$

The set of all local equilibria can be described in terms of properties of the directed graph with nodes $S_1,\ldots ,S_N$ and edges $S_i \rightarrow S_j$, when $k_{ji}>0$. Roughly speaking, there is a simple characterization of local equilibria, if the graph has enough edges.

#### Assumption A1

The directed graph corresponding to the reaction network is *connected* and *weakly reversible,* which means that for each pair $(i,j)\in \{1,\ldots ,N\}^2$ there exists a *path*$(j=i_0,i_1,\ldots ,i_{P_{ij}}=i)$ such that $k_{i_p i_{p-1}}>0$, $p=1,\ldots ,P_{ij}$.

**Fig. 1:**
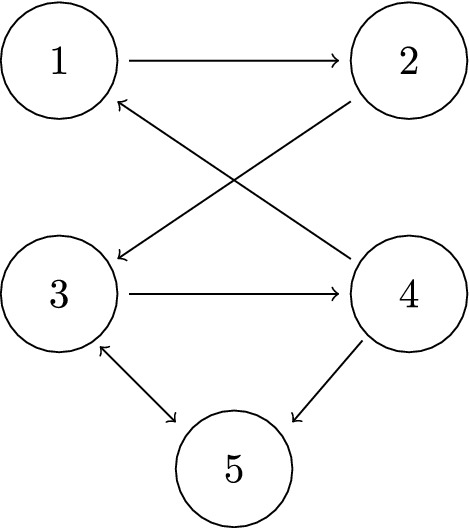
A connected and weakly reversible reaction network. Examples for shortest path lengths: $P_{14} = 1$, $P_{52} = 2$, $P_{25} = 4$, the latter with the path (5, 3, 4, 1, 2)

An example is given in Fig. 1. Note that the path from *j* to *i* is in general not unique. For the following a fixed choice of a path of minimal length $P_{ij}$ is used for each pair (*i*, *j*). This also means that paths are not self-intersecting in the sense that each reaction $S_{i_{p-1}}\rightarrow S_{i_p}$ appears only once.

#### Lemma 2

Let Assumption A1 hold. Then every local equilibrium is of the form $\rho (x,t)F(v)$ with

$$\begin{aligned} F_i(v) = \eta _i M_i(v) \,,\quad i = 1,\ldots ,N \,, \end{aligned}$$

where $\rho (x,t)$ is arbitrary and $(\eta _1,\ldots ,\eta _N)$ is the unique solution of

11 $$\begin{aligned} \sum _{j=1}^{N}\big (k_{ij}\eta _j - k_{ji}\eta _i\big ) = 0 \,,\quad i = 1,\ldots ,N \,,\qquad \sum _{i=1}^N \eta _i = 1 \,, \end{aligned}$$

satisfying $$\eta _i > 0$$, $$i=1,\ldots ,N$$.

#### Proof

A first consequence of Assumption A1 is that for each $i\in \{1,\ldots ,N_l\}$ there exists at least one $k_{ji}>0$ and at least one $k_{ij}>0$. This implies that for a local equilibrium $f_i(x,v,t) = \rho _i(x,t)M_i(v)$, $i=1,\ldots ,N_l$, must hold. Therefore

$$\begin{aligned} \sum _{j=1}^{N}\big (k_{ij}\rho _j - k_{ji}\rho _i\big ) =0\,,\quad i=1,\ldots ,N \,. \end{aligned}$$

Now it is a standard result of reaction network theory (see, e.g., [8, 12]), in our simple case of first order reactions related to the Perron-Frobenius theorem, that the connectedness and weak reversibility imply that there is a one-dimensional solution space spanned by $(\eta _1,\ldots ,\eta _N)$, where all components have the same sign. In the language of reaction network theory these are *complex balanced equilibria*. $\square$

A *global equilibrium* is a local equilibrium, which is also a steady state solution of (4), (5) compatible with conservation of total mass. Since at least one equation has a transport term, the function $\rho$ from Lemma 2 has to be constant for a global equilibrium. In the case $\Omega ={\mathbb {R}}^d$ we expect dispersion and consequential decay to zero. Therefore a nontrivial global equilibrium is only defined for $\Omega ={\mathbb {T}}^d$ by

12 $$\begin{aligned} f_\infty (x,v) := \rho _\infty F(v) \,, \qquad \text {with } \rho _\infty := \mathbf{M} /L^d \,. \end{aligned}$$

### Microscopic Coercivity—Convergence to Equilibrium

We write the system (7) in the abstract form

13 $$\begin{aligned} \partial _t f + \mathsf {T}f = \mathsf {L}f \,, \end{aligned}$$

with the *transport operator*$\mathsf {T}$ and the *reaction operator*$\mathsf {L}$ defined as

14 $$\begin{aligned} (\mathsf {T}f)_i = \sigma _i\, v\cdot \nabla _x f_i \,, \qquad (\mathsf {L}f)_i = \sum _{j=1}^{N}\big (k_{ij}\rho _j M_i - k_{ji}f_i \big ) \,,\qquad i=1,\ldots N\,. \end{aligned}$$

Lemma 2 characterizes the nullspace of the reaction operator. A projection to this nullspace is given by

15 $$\begin{aligned} \Pi f := \rho F \,,\qquad \text {with } \rho = \sum _{j=1}^{N}\rho _j \,. \end{aligned}$$

It is easily seen that $\Pi$ is a projection and that $\langle \Pi f,g\rangle = \langle \Pi f,\Pi g\rangle$, which implies that $\Pi$ is orthogonal. Since the application of the projection involves integration with respect to *v* and summation over all species, we do not only have $\mathsf {L}\Pi =0$, but the mass conservation property of the collision operator can be written as $\Pi \mathsf {L}= 0$.

Considering the quadratic relative entropy with respect to the local equilibrium *F* suggests the introduction of the weighted $L^2$-space ${{\mathcal {H}}}$ with the scalar product

16 $$\begin{aligned} \langle f,g \rangle := \sum _{i=1}^N \int _{\Omega }\int _{{\mathbb {R}}^d} \frac{f_i g_i}{F_i} dv\,dx \,, \end{aligned}$$

and with the induced norm $\Vert \cdot \Vert$. In the case $\Omega ={\mathbb {T}}^d$, the members of ${{\mathcal {H}}}$ are periodic with respect to *x*. Note that, for $i>N_l$, we have $f_i(x,v) = \rho _{f,i}(x)\delta (v)$, $g_i(x,v) = \rho _{g,i}(x)\delta (v)$, $F_i(x,v) = \eta _i\delta (v)$, and it has to be understood that

$$\begin{aligned} \int _{\Omega }\int _{{\mathbb {R}}^d} \frac{f_i g_i}{F_i} dv\,dx = \int _{\Omega }\int _{{\mathbb {R}}^d}\frac{\rho _{f,i}(x)\rho _{g,i}(x)}{\eta _i} \delta (v) dv\,dx = \int _{\Omega } \frac{\rho _{f,i}(x)\rho _{g,i}(x)}{\eta _i} dx,\quad i> N_l\,. \end{aligned}$$

Our main technical result, which will be proved in the following section, is coercivity of the reaction operator with respect to its null space. This property will be called *microscopic coercivity.*

#### Lemma 3

Let Assumption A1 hold. Then $\Pi$ defined by (15) is the orthogonal projection to the nullspace of the reaction operator $\mathsf {L}: {{\mathcal {H}}}\rightarrow {{\mathcal {H}}}$ defined in (14). Furthermore there exists a constant $\lambda _m>0$ such that

$$\begin{aligned} -\langle \mathsf {L}f,f \rangle \ge \lambda _m \Vert (1-\Pi )f\Vert ^2 \,. \end{aligned}$$

This lemma is one of the main tools in the proofs of our results on the long time behavior, presented in Sect. 4:

#### Theorem 4

Let Assumption A1 hold and let $\Omega ={\mathbb {T}}^d$. Then there exist constants $C,\lambda >0$ such that for every $f_I\in {{\mathcal {H}}}$ and with $f_\infty$ given by (12), the solution *f* of (7), (8) satisfies

$$\begin{aligned} \Vert f(\cdot ,\cdot ,t) - f_\infty \Vert ^2 \le Ce^{-\lambda t} \Vert f_I - f_\infty \Vert ^2 \,. \end{aligned}$$

#### Theorem 5

Let Assumption A1 hold and let $\Omega ={\mathbb {R}}^d$. Then for every $f_I\in {{\mathcal {H}}}\cap L^1(dv\,dx)$ there exists a constant $C>0$ such that the solution *f* of (7), (8) satisfies

$$\begin{aligned} \Vert f(\cdot ,\cdot ,t)\Vert ^2 \le C(1+t)^{-d/2}\,. \end{aligned}$$

### Macroscopic (Fast Reaction) Limit

We introduce a diffusive macroscopic rescaling $x\rightarrow x/\varepsilon$, $t\rightarrow t/\varepsilon ^2$ with $0<\varepsilon \ll 1$. Note, however, that in the case $\Omega ={\mathbb {T}}^d$ we still consider a fixed $\varepsilon$-independent torus after the rescaling. The abstract form (13) of our system becomes

17 $$\begin{aligned} \varepsilon ^2 \partial _t f_\varepsilon + \varepsilon \mathsf {T}f_\varepsilon = \mathsf {L}f_\varepsilon \,, \end{aligned}$$

with a now $\varepsilon$-dependent solution $f_\varepsilon$. Assuming convergence to $f_0$ as $\varepsilon \rightarrow 0$, the formal limit of the equation implies that $f_0$ is a local equilibrium, i.e. $f_0(x,v,t) = (\Pi f_0)(x,v,t) = \rho _0(x,t)F(v)$. It remains to determine $\rho _0$. The rescaled microscopic part $R_\varepsilon := \frac{(1-\Pi )f_\varepsilon }{\varepsilon }$ satisfies

18 $$\begin{aligned} \varepsilon \partial _t f_\varepsilon + \mathsf {T}f_\varepsilon = \mathsf {L}R_\varepsilon \end{aligned}$$

with the formal limit

19 $$\begin{aligned} \mathsf {T}f_0 = \mathsf {L}R_0 \,. \end{aligned}$$

Finally, we also need the conservation law

$$\begin{aligned} \partial _t \Pi f_\varepsilon + \Pi \mathsf {T}\frac{f_\varepsilon }{\varepsilon } = \partial _t \Pi f_\varepsilon + \Pi \mathsf {T}\frac{\Pi f_\varepsilon }{\varepsilon } + \Pi \mathsf {T}R_\varepsilon = 0 \,, \end{aligned}$$

and observe that the diffusive scaling is consistent, since the *diffusive macroscopic limit* identity

20 $$\begin{aligned} \Pi \mathsf {T}\Pi = 0 \end{aligned}$$

holds, which is easily checked, since $\Pi$ maps to a vector of centered Maxwellians, $\mathsf {T}$ provides a factor *v*, and the second application of $\Pi$ involves an integration with respect to *v*. The property (20) will also be essential in the proof of decay to equilibrium in Sect. 4 and it guarantees the necessary solvability condition $\Pi \mathsf {T}f_0 = 0$ for (19). Substituting its solution into the limiting conservation law should provide the missing information on $\rho _0$:

21 $$\begin{aligned} \partial _t \Pi f_0 + \Pi \mathsf {T}{\widehat{\mathsf {L}}}^{-1}\mathsf {T}f_0 = 0 \,, \end{aligned}$$

where ${\widehat{\mathsf {L}}}$ denotes the restriction of $\mathsf {L}$ to $(1-\Pi ){{\mathcal {H}}}$.

In order to translate the abstract result, we first note that

$$\begin{aligned} (\mathsf {T}\Pi f_0)_i = \sigma _i \eta _i M_i \,v\cdot \nabla _x\rho _0 \,,\qquad i=1,\ldots ,N \,. \end{aligned}$$

Since application of $\Pi$ involves integration with respect to *v* as first step, the identity $\int _{{\mathbb {R}}^d} vM_i \,dv = 0$ implies (20). The solution of (19) is then given by

$$\begin{aligned} R_{0,i} = \left( {\widehat{\mathsf {L}}}^{-1}\mathsf {T}f_0 \right) _i = - \frac{\eta _i M_i}{K_i} v\cdot \nabla _x\rho _0 \,,\qquad K_i = \sum _{j=1}^{N}k_{ji} \,, \qquad i\le N_l \,. \end{aligned}$$

A straightforward computation gives

$$\begin{aligned} \Pi \mathsf {T}{\widehat{\mathsf {L}}}^{-1}\mathsf {T}f_0 = -D\Delta _x \rho _0 \,F \,,\qquad D = \sum _{i=1}^{N_l} \frac{\eta _i \theta _i}{K_i} \,. \end{aligned}$$

Thus, (21) is equivalent to the diffusion equation

22 $$\begin{aligned} \partial _t \rho _0 = D\Delta _x \rho _0 \,. \end{aligned}$$

The following result, providing a rigorous justification of the formal asymptotics, will be proved in Sect. 5. It also relies on the microscopic coercivity result Lemma 3.

#### Theorem 6

Let Assumption A1 and $f_I\in {{\mathcal {H}}}$ hold (with $\Omega ={\mathbb {R}}^d$ or $\Omega ={\mathbb {T}}^d$). Then the solution $f_\varepsilon$ of (8), (17) converges, as $\varepsilon \rightarrow 0+$, to $\rho _0 F$ in $L_{loc}^\infty ({\mathbb {R}}^+;{{\mathcal {H}}})$ weak *, where $\rho _0\in L^\infty ({\mathbb {R}}^+;L^2(\Omega ))$ is a distributional solution of (22) subject to the initial condition $\rho _0(x,0) = \int _{{\mathbb {R}}^d}f_I(x,v)\,dv\;$.

## Microscopic Coercivity (Proof of Lemma 3)

We shall give two different proofs of the coercivity result. Both are inspired by the proof of the corresponding result in [8]. The first approach is based on a splitting between the species and velocity spaces, where for the former the result of [8] can be used directly. In the second approach the reaction paths of Assumption A1 are extended to paths in the larger species-velocity space. In both cases the coercivity constant $\lambda _m$ can in principle be computed explicitly. Since the computations are rather based on algorithms than on explicit formulas, a comparison of the results for both approaches would be difficult.

For the following computations we introduce $U_i := \frac{f_i}{\eta _i M_i}$, $V_i := \frac{g_i}{\eta _i M_i}$ and rewrite the reaction operator as

$$\begin{aligned} \mathsf {L}f = \sum _{j=1}^{N}M_i \left( k_{ij} \eta _j \int M_j' U_j' dv' - k_{ji}\eta _i U_i\right) \,, \end{aligned}$$

where the primes indicate evaluation at $v'$. This implies

$$\begin{aligned} \langle \mathsf {L}f,g\rangle= & {} \sum _{i,j=1}^N k_{ij} \eta _j \int _{\Omega \times {\mathbb {R}}^d\times {\mathbb {R}}^d} M_i M_j' U_j' V_i \,dv'\,dv\,dx -\frac{1}{2} \sum _{i,j=1}^N k_{ji} \eta _i \int _{\Omega \times {\mathbb {R}}^d} M_i U_i V_i \,dv\,dx\\&-\frac{1}{2} \sum _{i,j=1}^N k_{ji} \eta _i \int _{\Omega \times {\mathbb {R}}^d\times {\mathbb {R}}^d} M_i M_j' U_i V_i \,dv'\,dv\,dx \,. \end{aligned}$$

Now (11) is used in the second term on the right hand side and the change of variables $(i,v)\leftrightarrow (j,v')$ in the third:

$$\begin{aligned} \langle \mathsf {L}f,g\rangle = -\frac{1}{2} \sum _{i,j=1}^N k_{ij} \eta _j \int _{\Omega \times {\mathbb {R}}^d\times {\mathbb {R}}^d} M_i M_j' \left( U_i V_i + U_j' V_j' - 2 U_j' V_i\right) dv'\,dv\,dx \end{aligned}$$

This shows that $\mathsf {L}$ can only be expected to be symmetric in the case of *detailed balance*, i.e. when $k_{ij}\eta _j = k_{ji}\eta _i$ for all $i,j=1,\ldots ,N$. It also shows negative semi-definiteness of $\mathsf {L}$:

23 $$\begin{aligned} \langle \mathsf {L}f,f\rangle = -\frac{1}{2} \sum _{i,j=1}^N k_{ij}\eta _j \int _{\Omega \times {\mathbb {R}}^d\times {\mathbb {R}}^d} M_i M_j' (U_i - U_j')^2\,dv'\,dv\,dx \le 0 \,. \end{aligned}$$

**First proof – separation of species and velocity contributions:** The strategy is to split the dissipation term into contributions measuring the deviation from Maxwellian velocity distributions on the one hand, and from reaction equilibria of the position densities on the other hand:

24 $$\begin{aligned} - \langle \mathsf {L}f,f\rangle= & {} \frac{1}{2} \sum _{i,j=1}^N k_{ij}\eta _j \int _{\Omega \times {\mathbb {R}}^d\times {\mathbb {R}}^d} M_i M_j' \left[ U_i - \frac{\rho _i}{\eta _i} + \frac{\rho _i}{\eta _i} - \frac{\rho _j}{\eta _j} + \frac{\rho _j}{\eta _j} - U_j'\right] ^2\,dv'\,dv\,dx \nonumber \\= & {} \sum _{i=1}^{N}\left( \sum _{j=1}^{N}\frac{k_{ij} \eta _j^2 + k_{ji} \eta _i^2}{2\eta _i\eta _j}\right) \int _{\Omega \times {\mathbb {R}}^d} \frac{(f_i - \rho _i M_i)^2}{\eta _i M_i} dv\,dx \nonumber \\&+ \sum _{i,j=1}^{N}\frac{k_{ij}\eta _j}{2} \int _{\Omega } \left( \frac{\rho _i}{\eta _i} - \frac{\rho _j}{\eta _j}\right) ^2 dx \,. \end{aligned}$$

The norm of the microscopic part of the distribution is split correspondingly:

25 $$\begin{aligned} \Vert (1-\Pi )f\Vert ^2= & {} \sum _{i=1}^{N}\int _{\Omega \times {\mathbb {R}}^d} \frac{(f_i - \rho _i M_i + \rho _i M_i - \rho \eta _i M_i)^2}{\eta _i M_i} dv\,dx \nonumber \\= & {} \sum _{i=1}^{N}\int _{\Omega \times {\mathbb {R}}^d} \frac{(f_i - \rho _i M_i)^2}{\eta _i M_i} dv\,dx + \sum _{i=1}^{N}\int _{\Omega } \frac{(\rho _i - \rho \eta _i)^2}{\eta _i} dx \,. \end{aligned}$$

On the one hand the connectedness of the reaction network implies

$$\begin{aligned} \min _{1\le i\le N} \sum _{j=1}^{N}\frac{k_{ij} \eta _j^2 + k_{ji} \eta _i^2}{2\eta _i\eta _j} =: \gamma _1 > 0 \,, \end{aligned}$$

which allows to relate the first terms on the right hand sides of (24) and (25). On the other hand [8, eqn. (2.15)] could be used for the second terms. For comparability of the results of the two proofs we include the derivation of this second inequality. We start with the relation

$$\begin{aligned} \sum _{i=1}^{N}\int _\Omega \frac{(\rho _i - \rho \eta _i)^2}{\eta _i} dx = \frac{1}{2} \sum _{i,j=1}^{N}\eta _i\eta _j \int _\Omega \left( \frac{\rho _i}{\eta _i} - \frac{\rho _j}{\eta _j}\right) ^2 dx \,, \end{aligned}$$

which is easily verified by adding and subtracting $\rho$ in the parenthesis on the right hand side, expanding the square, and using that $\rho$ is the total density. For each pair (*i*, *j*) we use Assumption A1 and choose a path of minimal length $P_{ij}$ from *j* to *i*, which implies

$$\begin{aligned} \sum _{i=1}^{N}\int _\Omega \frac{(\rho _i - \rho \eta _i)^2}{\eta _i} dx= & {} \frac{1}{2} \sum _{i,j=1}^{N}\eta _i\eta _j \int _\Omega \left( \sum _{p=1}^N \left( \frac{\rho _{i_p}}{\eta _{i_p}} - \frac{\rho _{i_{p-1}}}{\eta _{i_{p-1}}}\right) \right) ^2 dx \\\le & {} \frac{1}{2} \sum _{i,j=1}^{N}\eta _i\eta _j P_{ij} \sum _{p=1}^N \int _\Omega \left( \frac{\rho _{i_p}}{\eta _{i_p}} - \frac{\rho _{i_{p-1}}}{\eta _{i_{p-1}}}\right) ^2 dx \,, \end{aligned}$$

by an application of the Cauchy-Schwarz inequality. With the definition

$$\begin{aligned} \mu _{ij} := \min _{1\le p \le P_{ij}} k_{i_p i_{p-1}}\eta _{i_{p-1}} > 0\,, \end{aligned}$$

we have the further estimate

$$\begin{aligned} \sum _{i=1}^{N}\int _\Omega \frac{(\rho _i - \rho \eta _i)^2}{\eta _i} dx\le & {} \frac{1}{2} \sum _{i,j=1}^{N}\frac{\eta _i\eta _j P_{ij}}{\mu _{ij}} \sum _{p=1}^N k_{i_p,i_{p-1}}\eta _{i_{p-1}} \int _\Omega \left( \frac{\rho _{i_p}}{\eta _{i_p}} - \frac{\rho _{i_{p-1}}}{\eta _{i_{p-1}}}\right) ^2 dx \\\le & {} \frac{1}{\gamma _2} \sum _{i,j=1}^{N}\frac{k_{ij}\eta _j}{2} \int _\Omega \left( \frac{\rho _i}{\eta _i} - \frac{\rho _j}{\eta _j}\right) ^2 dx\,, \end{aligned}$$

with

26 $$\begin{aligned} \frac{1}{\gamma _2} := \sum _{i,j=1}^{N}\frac{\eta _i\eta _j P_{ij}}{\mu _{ij}} \,. \end{aligned}$$

In the second inequality above we have used that each pair $(i_p,i_{p-1})$ occurs only once in a reaction path of minimal length. This concludes the proof of microscopic coercivity with $\lambda _m = \min \{\gamma _1,\gamma _2\}$.

**Second proof – species-velocity space paths:** Starting from the representation (23) and using that the path from *j* to *i* is not self-intersecting we have

$$\begin{aligned} - \langle \mathsf {L}f,f\rangle\ge & {} \frac{\mu _{ij}}{2} \sum _{p=1}^{P_{ij}} \int _{\Omega \times {\mathbb {R}}^d\times {\mathbb {R}}^d} M_{i_p} M_{i_{p-1}}' (U_{i_p} - U_{i_{p-1}}')^2 \,dv'\,dv\,dx \\= & {} \frac{\mu _{ij}}{2} \sum _{p=1}^{P_{ij}} \int _{\Omega \times {\mathbb {R}}^{(P_{ij}+1)d}} \prod _{q=0}^{P_{ij}} M_{i_q}(v_q) \,(U_{i_p}(v_p) - U_{i_{p-1}}(v_{p-1}))^2 \,dv_0 \ldots dv_{P_{ij}}\,dx \,. \end{aligned}$$

With the Cauchy-Schwarz inequality on ${\mathbb {R}}^{P_{ij}}$ this can be estimated further by

27 $$\begin{aligned} - \langle \mathsf {L}f,f\rangle\ge & {} \frac{\mu _{ij}}{2P_{ij}} \int _{\Omega \times {\mathbb {R}}^{(P_{ij}+1)d}} \prod _{q=0}^{P_{ij}} M_{i_q}(v_q) \left( \sum _{p=1}^{P_{ij}} \left( U_{i_p}(v_p) - U_{i_{p-1}}(v_{p-1})\right) \right) ^2 \,dv_0 \ldots dv_{P_{ij}}\,dx \nonumber \\= & {} \frac{\mu _{ij}}{2P_{ij}} \int _{\Omega \times {\mathbb {R}}^{(P_{ij}+1)d}} \prod _{q=0}^{P_{ij}} M_{i_q}(v_q) \left( U_i(v_{P_{ij}}) - U_j(v_0)\right) ^2 \,dv_0 \ldots dv_{P_{ij}}\,dx \nonumber \\= & {} \frac{\mu _{ij}}{2P_{ij}} \int _{\Omega \times {\mathbb {R}}^{2d}} M_i M_j' \left( U_i - U_j'\right) ^2 \,dv'\,dv\,dx \,. \end{aligned}$$

As indicated at the beginning of this section, the strategy in these estimates was to extend the path $i_0,\ldots ,i_{P_{ij}}$ in the species space $\{1,\ldots ,N\}$ to all possible paths of the form $(i_0,v_0),\ldots , (i_{P_{ij}},v_{P_{ij}})$ in the species-velocity space $\{1,\ldots ,N\} \times {\mathbb {R}}^d$.

As the next step we observe that

$$\begin{aligned}&\sum _{i,j=1}^N \int _{\Omega \times {\mathbb {R}}^{2d}} \eta _i \eta _j M_i M_j' (U_i - U_j')^2 dv'\,dv\,dx \\&\quad = \sum _{i,j=1}^N \int _{\Omega \times {\mathbb {R}}^{2d}} \eta _i \eta _j M_i M_j' \left( (U_i - \rho )^2 + (U_j' - \rho )^2 - 2(U_i - \rho )(U_j' - \rho ) \right) dv'\,dv\,dx \\&\quad = 2\Vert (1-\Pi )f\Vert ^2 \,. \end{aligned}$$

Combining this with (27) completes the alternative proof of Lemma 3 with $\lambda _m = \gamma _2$, as defined in (26). The result of the second proof is always at least as good as that of the first.

## Hypocoercivity

Quantitative results on the decay to equilibrium will be shown by employing the hypocoercivity approach of [7] with modifications introduced in [2].

In the case of the torus, $\Omega ={\mathbb {T}}^d$, we assume w.l.o.g. $\rho _\infty = \mathbf{M} = 0$, which can always be achieved by a redefinition of the solution. Thus, in both cases $\Omega = {\mathbb {T}}^d$ and $\Omega = {\mathbb {R}}^d$ we expect $f\rightarrow 0$ as $t\rightarrow \infty$. The functional $f\mapsto \Vert f\Vert ^2$ can then be understood as relative entropy and a natural candidate for a Lyapunov function. However, by the obvious antisymmetry of the transport operator $\mathsf {T}$,

28 $$\begin{aligned} \frac{d}{dt} \frac{\Vert f\Vert ^2}{2} = \langle \mathsf {L}f , f \rangle \,, \end{aligned}$$

which is nonpositive as expected, but vanishes on the set of local equilibria, i.e. it lacks the definiteness required for a Lyapunov function.

In [7] a Lyapunov function, or modified entropy, $\mathsf {H}[f]$ has been proposed, which has the form

$$\begin{aligned} \mathsf {H}[f] := \frac{\Vert f\Vert ^2}{2} + \delta \langle \mathsf {A}f , f \rangle \,,\qquad \text{ with } \mathsf {A}:= [1 + (\mathsf {T}\Pi )^\star (\mathsf {T}\Pi )]^{-1} (\mathsf {T}\Pi )^\star \end{aligned}$$

and with a small parameter $\delta >0$ to be determined later. In [7, Lemma 1] it has been shown that the operator norm of $\mathsf {A}$ is bounded by $\frac{1}{2}$, such that

29 $$\begin{aligned} \frac{1 - \delta }{2} \Vert f\Vert ^2 \le \mathsf {H}[f] \le \frac{1 + \delta }{2} \Vert f\Vert ^2 \,, \end{aligned}$$

and $\mathsf {H}[f]$ is equivalent to $\Vert f\Vert ^2$ for $\delta < 1$.

For solutions *f* of (13), its time derivative is given by

30 $$\begin{aligned} \frac{d}{dt}\mathsf {H}[f]&= \langle \mathsf {L}f , f \rangle - \delta \langle \mathsf {A}\mathsf {T}\Pi f , f \rangle - \delta \langle \mathsf {A}\mathsf {T}(1-\Pi )f , f \rangle + \delta \langle \mathsf {T}\mathsf {A}f , f \rangle \nonumber \\&\quad + \delta \langle \mathsf {A}\mathsf {L}f , f \rangle + \delta \langle \mathsf {A}f , \mathsf {L}f \rangle \,. \end{aligned}$$

From the definition of $\mathsf {A}$ it is clear that the property $\mathsf {A}= \Pi \mathsf {A}$ holds. On the other hand, the conservation of total mass by the collision operator is equivalent to $\Pi \mathsf {L}= 0$, i.e. $\Pi$ also projects to the nullspace of $\mathsf {L}^*$. As a consequence, the last term above vanishes:

$$\begin{aligned} \langle \mathsf {A}f , \mathsf {L}f \rangle = \langle \Pi \mathsf {A}f , \mathsf {L}f \rangle = \langle \mathsf {A}f , \Pi \mathsf {L}f \rangle = 0 \,. \end{aligned}$$

In [7] this property is the consequence of the assumption that $\mathsf {L}$ is symmetric, which does not hold here, as noted in the previous section.

The first term on the right hand side of (30) controls the microscopic part $(1-\Pi )f$ of the distribution function. The second term is responsible for the macroscopic part:

### Lemma 7

With the above notation,

$$\begin{aligned} \langle \mathsf {A}\mathsf {T}\Pi f , f \rangle = \Vert \mathsf {T}\Pi g\Vert ^2 + \Vert (\mathsf {T}\Pi )^* \mathsf {T}\Pi g\Vert ^2 = {\overline{D}} \Vert \nabla _x \rho _g\Vert _2^2 + {\overline{D}}^2\Vert \Delta _x \rho _g\Vert _2^2\,, \end{aligned}$$

where ${\overline{D}} = \sum _{i\le N_l} \eta _i\theta _i$, $\Vert \cdot \Vert _2$ = $\Vert \cdot \Vert _{L^2(\Omega )}$, and $g=\rho _g F$ is given by

$$\begin{aligned} g = (1+ (\mathsf {T}\Pi )^* \mathsf {T}\Pi )^{-1} \Pi f \,,\qquad \text{ i.e. } \rho _g \text{ solves }\quad \rho _g - {\overline{D}} \Delta _x \rho _g = \rho _f\,. \end{aligned}$$

### Proof

The property $g = \Pi g$ is obvious from its definition. We use the abbreviation ${\mathcal {L}} := (\mathsf {T}\Pi )^* \mathsf {T}\Pi$ and compute

$$\begin{aligned} \mathsf {A}\mathsf {T}\Pi f = (1+{\mathcal {L}})^{-1} {\mathcal {L}} \Pi f = (1+{\mathcal {L}})^{-1} (1 + {\mathcal {L}} - 1)\Pi f = \Pi f - g = {\mathcal {L}}g \,. \end{aligned}$$

Therefore, using again the property $\mathsf {A}=\Pi \mathsf {A}$,

$$\begin{aligned} \langle \mathsf {A}\mathsf {T}\Pi f , f \rangle = \langle \mathsf {A}\mathsf {T}\Pi f , \Pi f \rangle = \langle {\mathcal {L}}g , g + {\mathcal {L}}g \rangle = \Vert \mathsf {T}\Pi g\Vert ^2 + \Vert {\mathcal {L}}g\Vert ^2 \,. \end{aligned}$$

A straightforward computation shows ${\mathcal {L}}g = -{\overline{D}} \Delta _x \rho _g\,F$, completing the proof. $\square$

As a consequence, the first two terms on the right hand side of (30) provide the desired definiteness, since obviously $\langle \mathsf {A}\mathsf {T}\Pi f , f \rangle \ge 0$ and $\langle \mathsf {A}\mathsf {T}\Pi f , f \rangle = 0\,\Rightarrow \, \rho _g=0\,\Rightarrow \,\Pi f = 0$. However, the remaining terms still need to be controlled.

We start by using the diffusive macroscopic limit property (20), implying

$$\begin{aligned} \mathsf {T}\mathsf {A}= -\mathsf {T}\Pi (1+{\mathcal {L}})^{-1} \Pi \mathsf {T}= -(1-\Pi )\mathsf {T}\Pi (1+{\mathcal {L}})^{-1} \Pi \mathsf {T}(1-\Pi ) \,. \end{aligned}$$

In [7, Lemma 1] it has been shown that the operator norm of $\mathsf {T}\mathsf {A}$ is bounded by 1 implying, together with the above,

31 $$\begin{aligned} |\langle \mathsf {T}\mathsf {A}f , f \rangle | \le \Vert (1-\Pi )f\Vert ^2 \,. \end{aligned}$$

### Lemma 8

With the above notation,

$$\begin{aligned} |\langle \mathsf {A}\mathsf {T}(1-\Pi )f , f \rangle | \le C_1 \Vert (1-\Pi )f\Vert \langle \mathsf {A}\mathsf {T}\Pi f , f \rangle ^{1/2} \qquad \text{ with } C_1 = \frac{1}{{\overline{D}}} \left( d(d+2)\sum _{i=1}^{N_l} \eta _i \theta _i^2\right) ^{1/2}\,. \end{aligned}$$

### Proof

With *g* as introduced in Lemma 7 and with $\mathsf {A}^* = \mathsf {T}\Pi (1+{\mathcal {L}})^{-1} = \mathsf {T}(1+{\mathcal {L}})^{-1}\Pi$ we have

$$\begin{aligned} \langle \mathsf {A}\mathsf {T}(1-\Pi )f , f \rangle = - \langle (1-\Pi )f , \mathsf {T}\mathsf {A}^*f \rangle = -\langle (1-\Pi )f , \mathsf {T}^2 g \rangle \end{aligned}$$

and

$$\begin{aligned} (\mathsf {T}^2 g)_i = \sigma _i \,v\cdot \nabla _x (v\cdot \nabla _x \rho _g)F_i \,. \end{aligned}$$

The estimate

$$\begin{aligned} \Vert \mathsf {T}^2 g\Vert ^2 \le \sum _{i=1}^{N_l} \int _{{\mathbb {R}}^d}|v|^4 F_i\,dv\; \Vert \nabla _x^2 \rho _g\Vert _2^2 = d(d+2)\sum _{i=1}^{N_l} \eta _i \theta _i^2 \,\Vert \Delta _x \rho _g\Vert _2^2 \end{aligned}$$

and an application of Lemma 7 complete the proof. $\square$

### Lemma 9

With the above notation,

$$\begin{aligned} |\langle \mathsf {A}\mathsf {L}f , f \rangle |\le & {} C_2 \Vert (1-\Pi )f\Vert \langle \mathsf {A}\mathsf {T}\Pi f , f \rangle ^{1/2} \qquad \text{ with } \\ C_2= & {} \left( 2N \max _{1\le j\le N} \sum _{i=1}^{N}\frac{k_{ij}^2}{\eta _i} + 2 \max _{1\le i\le N} \left( \sum _{j=1}^{N}k_{ji} \right) ^2\right) ^{1/2}\,. \end{aligned}$$

### Proof

We use (remembering $\mathsf {L}= \mathsf {L}(1-\Pi )$ and, from the preceding proof, $\mathsf {A}^* f = \mathsf {T}\Pi g$)

$$\begin{aligned} \langle \mathsf {A}\mathsf {L}f , f \rangle = \langle \mathsf {L}(1-\Pi )f , \mathsf {T}\Pi g \rangle \,, \end{aligned}$$

Lemma 7, and the boundedness of $\mathsf {L}$:

$$\begin{aligned} \Vert \mathsf {L}f\Vert ^2\le & {} 2 \sum _{i=1}^{N}\int _{{\mathbb {R}}^d}\int _\Omega \frac{M_i}{\eta _i} \left( \sum _{j=1}^{N}k_{ij}\rho _j\right) ^2 dx\,dv\; + 2 \sum _{i=1}^{N}\int _{{\mathbb {R}}^d}\int _\Omega \frac{f_i^2}{F_i} \left( \sum _{j=1}^{N}k_{ji}\right) ^2 dx\,dv\; \\\le & {} 2N \sum _{j=1}^{N}\sum _{i=1}^{N}\frac{k_{ij}^2}{\eta _i} \int _\Omega \rho _j^2 dx + 2 \max _{1\le i\le N} \left( \sum _{j=1}^{N}k_{ji} \right) ^2 \Vert f\Vert ^2 \\\le & {} \left( 2N \max _{1\le j\le N} \sum _{i=1}^{N}\frac{k_{ij}^2}{\eta _i} + 2 \max _{1\le i\le N} \left( \sum _{j=1}^{N}k_{ji} \right) ^2\right) \Vert f\Vert ^2 \,. \end{aligned}$$

$$\square $$

Collecting the results of Lemmas 3, 7, 8, and 9 , we obtain

$$\begin{aligned} \frac{d}{dt}\mathsf {H}[f] \le -(\lambda _m - \delta )\Vert (1-\Pi )f\Vert ^2 - \delta \langle \mathsf {A}\mathsf {T}\Pi f , f \rangle + \delta (C_1 + C_2)\Vert (1-\Pi )f\Vert \langle \mathsf {A}\mathsf {T}\Pi f , f \rangle ^{1/2} \,. \end{aligned}$$

Thus, for

$$\begin{aligned} \delta < \frac{4\lambda _m}{4+(C_1+C_2)^2} \,, \end{aligned}$$

we have

32 $$\begin{aligned} \frac{d}{dt}\mathsf {H}[f] \le - \lambda _\delta (\Vert (1-\Pi )f\Vert ^2 + \langle \mathsf {A}\mathsf {T}\Pi f , f \rangle ) \end{aligned}$$

with

$$\begin{aligned} \lambda _\delta = \frac{1}{2} \left( \lambda _m - \sqrt{\lambda _m^2 - \delta ( 4\lambda _m - 4\delta - \delta (C_1 + C_2)^2)}\right) >0 \,. \end{aligned}$$

This shows that $\mathsf {H}[f]$ is a Lyapunov function. It remains to obtain the decay rates.

### Exponential Decay on the Torus (Proof of Theorem 4)

For the case of the torus, i.e. $\Omega = {\mathbb {T}}^d$, recalling the normalization to $\rho _\infty =0$ from the beginning of this section, with the notation of Lemma 7 we have

$$\begin{aligned} \int _{{\mathbb {T}}^d} \rho _g \,dx = \int _{{\mathbb {T}}^d} \rho _f \,dx = 0 \,. \end{aligned}$$

The Poincaré inequality on the torus therefore provides *macroscopic coercivity*, i.e. there exists $\lambda _M>0$ such that

$$\begin{aligned} \Vert \mathsf {T}\Pi g\Vert ^2 = {\overline{D}} \Vert \nabla _x \rho _g\Vert _2^2 \ge \lambda _M \Vert \rho _g\Vert _2^2 = \lambda _M \Vert g\Vert ^2 \,. \end{aligned}$$

This is used in

$$\begin{aligned} \Vert \Pi f\Vert ^2= & {} \Vert g + {\mathcal {L}}g\Vert ^2 = \Vert g\Vert ^2 + 2\Vert \mathsf {T}\Pi g\Vert ^2 + \Vert {\mathcal {L}}g\Vert ^2 \le \left( \frac{1}{\lambda _M} + 2\right) \Vert \mathsf {T}\Pi g\Vert ^2 + \Vert {\mathcal {L}}g\Vert ^2 \\\le & {} \left( \frac{1}{\lambda _M} + 2\right) \langle \mathsf {A}\mathsf {T}\Pi f , f \rangle \,. \end{aligned}$$

Combining this estimate with (29) and with (32) gives

$$\begin{aligned} \frac{d}{dt} \mathsf {H}[f] \le - \frac{2\lambda _\delta \lambda _M}{(1+2\lambda _M)(1+\delta )} \mathsf {H}[f] \,, \end{aligned}$$

completing the proof of Theorem 4 with

$$\begin{aligned} \lambda = \frac{2\lambda _\delta \lambda _M}{(1+2\lambda _M)(1+\delta )} \,,\qquad C = \frac{1+\delta }{1-\delta } \,, \qquad \delta < \min \left\{ 1, \frac{4\lambda _m}{4+(C_1+C_2)^2}\right\} \,. \end{aligned}$$

### Algebraic Decay on the Whole Space (Proof of Theorem 5)

In the case $\Omega ={\mathbb {R}}^d$ macroscopic coercivity fails and is replaced by the Nash inequality [16]

$$\begin{aligned} \Vert u\Vert _2^{2+4/d} \le {\mathcal {C}} \Vert \nabla u\Vert _2^2 \Vert u\Vert _1^{4/d} \qquad \forall \, u \in H^1({\mathbb {R}}^d)\cap L^1({\mathbb {R}}^d) \,. \end{aligned}$$

Noting that

$$\begin{aligned} \int _{{\mathbb {R}}^d} \rho _g \,dx = \int _{{\mathbb {R}}^d} \rho _f \,dx = \mathbf{M} \,, \end{aligned}$$

it implies

$$\begin{aligned} \Vert \mathsf {T}\Pi g\Vert ^2 = {\overline{D}} \Vert \nabla _x \rho _g\Vert _2^2 \ge \kappa _M \Vert g\Vert ^{2+4/d} \,,\qquad \text{ with } \kappa _M = \frac{\overline{D}}{{\mathcal {C}}{} \mathbf{M} ^{4/d}} \,, \end{aligned}$$

and, thus,

$$\begin{aligned} \Vert \Pi f\Vert ^2\le & {} \kappa _M^{-\frac{d}{d+2}} \Vert \mathsf {T}\Pi g\Vert ^{\frac{2d}{d+2}} + 2\Vert \mathsf {T}\Pi g\Vert ^2 + \Vert {\mathcal {L}}g\Vert ^2 \\\le & {} \kappa _M^{-\frac{d}{d+2}}\langle \mathsf {A}\mathsf {T}\Pi f , f \rangle ^{\frac{d}{d+2}} + 2 \langle \mathsf {A}\mathsf {T}\Pi f , f \rangle =: \Phi (\langle \mathsf {A}\mathsf {T}\Pi f , f \rangle ) \,. \end{aligned}$$

Furthermore, by the properties of $\Phi$,

$$\begin{aligned} \Vert f\Vert ^2 \le \Phi \left( \Vert (1-\Pi )f\Vert ^2 + \langle \mathsf {A}\mathsf {T}\Pi f , f \rangle \right) \end{aligned}$$

Similarly to the case of the torus we now obtain from (32) the differential inequality

$$\begin{aligned} \frac{d}{dt} \mathsf {H}[f] \le -\lambda _\delta \Phi ^{-1}\left( \frac{2}{1+\delta } \mathsf {H}[f]\right) \,. \end{aligned}$$

The decay of $\mathsf {H}[f]$ can be estimated by the solution *z* of the corresponding ODE problem

$$\begin{aligned} \frac{dz}{dt} = - \lambda _\delta \Phi ^{-1}\left( \frac{2z}{1+\delta } \right) \,,\qquad z(0) = \mathsf {H}[f_I] \,. \end{aligned}$$

By the properties of $\Phi$ it is obvious that $z(t)\rightarrow 0$ monotonically as $t\rightarrow \infty$, which implies that the same is true for $\frac{dz}{dt}$. Therefore, there exists $t_0>0$ such that, in the rewritten ODE

$$\begin{aligned} \left( -\frac{2}{\lambda _\delta \kappa _M} \frac{dz}{dt}\right) ^{\frac{d}{d+2}} -\frac{2}{\lambda _\delta } \frac{dz}{dt} = \frac{2z}{1+\delta } \,, \end{aligned}$$

the second term is smaller than the first for $t\ge t_0$, implying the differential inequality

$$\begin{aligned} \frac{dz}{dt} \le -\kappa z^{1+2/d} \qquad \text{ for } t\ge t_0\,, \end{aligned}$$

with an appropriately defined positive constant $\kappa$. Integration gives

$$\begin{aligned} z(t) \le \left( \mathsf {H}[f_I]^{-2/d} + \frac{2\kappa }{d} t\right) ^{-d/2} \,, \end{aligned}$$

completing the proof of Theorem 5.

## The Rigorous Macroscopic Limit (Proof of Theorem 6)

The goal of this section is to justify the formal macroscopic limit $\varepsilon \rightarrow 0$, carried out in Sect. 2 on the scaled equation (17). The entropy dissipation relation (28) now reads

33 $$\begin{aligned} \frac{\varepsilon ^2}{2}\frac{d}{dt}\Vert f_\varepsilon \Vert ^2 = \langle \mathsf {L}f_\varepsilon , f_\varepsilon \rangle \,. \end{aligned}$$

Integration with respect to time and microscopic coercivity (Lemma 3) give

$$\begin{aligned} \frac{\varepsilon ^2}{2}\Vert f_\varepsilon (\cdot ,\cdot ,t)\Vert ^2 + \lambda _m \int _0^t \Vert (1-\Pi )f_\varepsilon (\cdot ,\cdot ,s)\Vert ^2 ds \le \frac{\varepsilon ^2}{2} \Vert f_I\Vert ^2 \,. \end{aligned}$$

From this relation we deduce that $f_\varepsilon$ is bounded in $L^\infty ({\mathbb {R}}^+;{{\mathcal {H}}})$ and that $R_\varepsilon = \frac{1}{\varepsilon }(1-\Pi )f_\varepsilon$ is bounded in $L^2({\mathbb {R}}^+;{{\mathcal {H}}})$, both uniformly as $\varepsilon \rightarrow 0$. As a consequence of the former, $\rho _\varepsilon = \int _{{\mathbb {R}}^d}f_\varepsilon \,dv\;$ is uniformly bounded in $L^\infty ({\mathbb {R}}^+;L^2({\mathbb {R}}^d))$. Therefore there exist weak accumulation points $f_0$, $R_0$, and $\rho _0$ of, respectively, $f_\varepsilon$, $R_\varepsilon$, and $\rho _\varepsilon$, satisfying $f_0(x,v,t) = \rho _0(x,t)F(v)$. These facts allow to pass to the limit in (18) and in the rescaled conservation law (9), i.e.

$$\begin{aligned} \varepsilon \partial _t f_\varepsilon + \mathsf {T}f_\varepsilon = \mathsf {L}R_\varepsilon \,,\qquad \partial _t \rho _\varepsilon + \nabla _x\cdot \sum _{i=1}^{N_l} \int _{{\mathbb {R}}^d}v R_{\varepsilon ,i}\,dv\; = 0 \,, \end{aligned}$$

in the sense of distributions. The limiting equations are equivalent to the distributional formulation of the heat equation (22) for $\rho _0$ with the initial condition $\rho _0(t=0) = \int _{{\mathbb {R}}^d}f_I\,dv\;$. The uniqueness of the solution of this problem implies the weak convergence of $f_\varepsilon$ to $\rho _0 F$. This completes the proof of Theorem 6.
